# Dr. Bushnan's Observations on Dysmenorrhœa

**Published:** 1840-10

**Authors:** 


### DYSMENORRHŒA.

[It gives us much pleasure to be able to insert in our pages the following letter which does equal honour to the professional zeal and the benevolence of its talented author. As the letter speaks for itself, we will only here request the best attention of our readers to it, and beg them to understand that, although Dr. Bushnan has addressed it especially to his brethren of the Provincial Association, he will be equally gratified by communications in answer to his appeal, from any members of the profession.]

*Observations on Dysmenorrhœa and its Treatment, addressed to the Members of the Provincial Medical Association.* By J. STEVENSON BUSHNAN, M.D., Fellow of the Royal College of Physicians of Edinburgh.

Gentlemen,—I am induced to address to you the observations and queries contained in the following circular, by the conviction that medical authorities have hitherto directed much less of their attention to dysmenorrhœa or difficult menstruation, than the frequency of the disease and its very injurious influence on female health imperatively call for.

My own attention has been strongly drawn of late to this affection by the very evident good effects of an ointment of veratria applied to the region of the sacrum; and it occurred to me, considering how slow and liable to fallacy the experience of a single individual is, even under the most favorable circumstances, that, by appealing to the zeal and humanity of my medical brethren, and particularly by availing myself of the good fellowship of the members of the Association, I might be able to collect, in a comparatively short time, with a view to publication, much information of the greatest practical use, regarding the varieties of this painful affection, the comparative frequency of each variety, and the extent to which in these the proposed remedy is applicable.

Since I stated the result of my experience of this remedy to the Provincial Medical and Surgical Association, at the Southampton Meeting in July, I have received numerous communications upon the subject, some asking for further information as to the employment of the ointment, and others announcing the complete success they have experienced by its use. From these, however, I shall select but one example: Dr. Jeffreys, of Liverpool, having determined to try the remedy, writes me on the 19th of August, "A most distinct and distressing instance has within these few last days fallen within the sphere of my observation; and the efficacy of your remedy has afforded me great satisfaction and unspeakable delight to the young lady and her family, whose age is about twenty, and who has suffered beyond all description from this distressing visitor, from the first appearance of the catamenia."

The ointment used was of the same strength as that which I employ, namely, made with half a drachm of the veratria to an ounce of prepared lard; and a portion the size of a hazel-nut was rubbed three or four times a day upon the sacrum.

That there should be a very considerable diversity in the symptoms as well as in the states of the habit in which dysmenorrhœa occurs, could not but be anticipated, even if the disease had been determined to be on the whole of one character. And it appears to me to be the greatest defect in our knowledge of this malady that it is not sufficiently settled whether the varieties are of an essential or only of an accidental kind, that is, whether there be forms of the disease which, as requiring from their own intrinsic nature a separate kind of treatment, deserve to be distinguished specifically from the others. Mere complications of a disease do not afford an adequate ground for the distinction of species: and thus, though it may be admitted that inflammatory symptoms do sometimes attend dysmenorrhœa, it does not therefore follow that the disease is ever essentially inflammatory or that there is an inflammatory species, that is, one depending for its existence on inflammation. It is, however, very desirable to have it settled beyond all doubt, whether there be such an inflammatory species, however rare, and if there be not, how far inflammatory symptoms are apt to be accidentally concomitant. Were it not for the statements of authors worthy of credit, I should be myself inclined to think that there is no essentially inflammatory species; and that inflammation, as a concomitant, is exceedingly rare.

Though it does not appear that the varieties commonly met with in practice, or described by authors, are of such a kind as not to be explicable on the supposition of the mere casual variation of one disease, still it is advisable, to ensure accuracy, to consider well any evidence that may be suggested to the contrary: and, in particular, to note under what difference of circumstances, if any, the simple and membranous discharges occur; again, to take pains to discover whether the violent and painful efforts which sometimes occur at the monthly periods, without any menstrual secretion whatever, and which has been termed menstrual ischuria, be more allied in their origin to dysmenorrhœa, or to amenorrhœa, to which last verbally they belong; also, whether there be anything peculiar in that form of the disease in which the secretion comes off painfully, drop by drop, named like the last, in analogy to the corresponding diseases of the urinary system, Menstrual Strangury. (Capuron, "Traité des Maladies des Femmes," p. 70.) Such names, however, I think it were better to avoid.

Moreover there are differences in the kind of pain in the time of its occurrence and of its duration which deserve attention, as possibly bearing on the character of each case. Thus, as it is well known, the pain usually precedes the discharge and disappears as this becomes free; yet on other occasions, the pain during its whole continuance, is attended with a discharge more copious than usual, escaping in gushes and mixed with coagulated blood, while at other times the pain does not begin till the secretion has gone on regularly for some time, the pain bringing with it either an increase or diminution of the discharge, but not ceasing till the secretion is entirely at an end. (Cyclop. Pract. Med.—Art. Dysmenorrhœa.)

Another point upon which some doubt seems to exist is, how far dysmenorrhœa is a cause of abortion, or whether it is not more uniformly a source of sterility. Unquestionably the membranous discharges have been mistaken for abortions; the proof however of such a mistake having been committed does not put the question at rest. This then is a part of the history of the disease still open to investigation; for there is no better agreement in regard to it among the authorities of our day than among those of the preceding ages. (See Parr, Lond. Med. Dic.—Art. Menses; Hamilton, Outlines of Midwifery; Cyclopædia, l. c.) I am myself inclined to think that conception is a very rare occurrence during the continuance of dysmenorrhœa.

Lastly, since leucorrhœa sometimes is conjoined with dysmenorrhœa, it is very desirable to ascertain as far as possible in what proportion of cases this complication exists.

The acknowledged good effects of opiates point to the nervous system as the source of this disease. It may be regarded as falling under what Andral terms, Perversion of Nervous Action. (Précis d'Anatomie Pathologique, tom. i. p. 574.) The vascular orgasm, or hyperemia of the uterine system which, occurring monthly, relieves itself by secretion in healthy females without any very great constitutional disturbance, proves too great a stimulus to the nervous system at large, or at least to that part of it connected with the female reproductive organs in those in whom an undue nervous excitability exists. Hence, as in other cases of unusual irritation acting on the nerves of a part, or of ordinary excitants operating on an unusual susceptibility of the same, extraordinary sensations and movements arise, and are followed by impediments to the natural function of the uterus, even in so far as it depends on the vascular system, in which the nerves themselves may be but remotely concerned. Narcotic substances, by diminishing the excitability of the nerves, break or weaken the chief link in this chain of morbid changes, and thus permit the ordinary powers of the system to operate with less embarrassment.*

* Any lengthened remarks upon the general theory of nervous diseases would be out of place; but, "brevis esse laboro, obscurus fio;" and I may here observe:

1. That unusual irritations of sentient nerves are followed by deranged actions of muscular organs through their motor nerves.

2. That ordinary stimuli acting on an unusual susceptibility of sentient nerves produce similar derangements.

3. That the nerves of the uterus are unusually susceptible of irritation at the menstrual periods.

4. That derangements of nervous action disturb secretion, even though secretion be not directly dependent on the nerves—being a function of the vascular system only.

Supposing this view to be correct, experience alone can teach what narcotics and what mode of applying them are best adapted to the purpose.

The benefit afforded by opium is undeniable; yet that does not exclude the possibility of the ointment of veratria being of much greater effect; and the application of it to the region of the sacrum appears to afford the readiest channel by which the pelvic nerves can be acted on.

My recommendation, however, of this remedy is not founded on speculation, but on experience; and I have a just confidence that success will also attend the employment of it in your practice, if you will consent to make the trial.

May I then request you to take the trouble to mark the result of your experience on the inclosed table and transmit it to me. Your doing so will confer on me a great obligation, and contribute, as I anticipate, to the diffusion of a more exact knowledge, among the profession at large, of the nature and treatment of this painful and distressing malady.

I have the honour to be, your most obedient servant,
J. Stevenson Bushnan.

Ansford House, Castle Cary, Somerset; Sept. 1840.

*Form of Table to be returned to* Dr. Bushnan *when filled up.*

